# Exploring why young Australians participate in the sport of fencing: Future avenues for sports‐based health promotion

**DOI:** 10.1002/hpja.650

**Published:** 2022-08-30

**Authors:** Elly Ganakas, Amy E. Peden

**Affiliations:** ^1^ School of Population Health University of New South Wales Kensington NSW Australia

**Keywords:** adolescents, health promotion strategies, physical activity, qualitative methods, sports

## Abstract

**Issue addressed:**

Australian sport policy calls for a diverse sector to get more individuals more active. This study contributes to an evidence base of alternative sport options that may increase physical activity levels in adolescents, as we sought to understand why Australians decide to participate in fencing when aged <18 years.

**Methods:**

A retrospective, mixed‐methods survey design was employed to measure why a participant started fencing and what participants like about fencing compared to other sports. Free‐text qualitative data were analysed thematically. Quantitative data were collected to answer secondary objectives, using nonparametric tests to determine significance between the median scores prefencing and postfencing for enjoyment and frequency of participation.

**Results:**

One hundred and one fencers across Australia who started fencing aged <18 years completed the survey. Respondents primarily started fencing for fun/enjoyment, followed by the desire to try something different/alternative/nonmainstream. Four themes were identified relating to why a participant decided to start fencing: (i) external motivators, (ii) influence of interest and imagination, (iii) developing the self and (iv) the supportive culture.

**Conclusions:**

Fencing may encourage adolescent participation in physical activity due to unique characteristics which attracts individuals for reasons beyond the need to be active alone, and instead affords a space for self‐exploration, learning, and belonging. Further research is required to identify how fencing can engage different cohorts of adolescents, and any barriers to participation.

**So what?:**

Fencing could be utilised to promote engagement in physical activity among Australian adolescents by emphasising the creative, mental, and inclusive aspects of the sport.

## 1 INTRODUCTION

The physical and mental health benefits derived from physical activity during childhood and adolescence are well‐established and include improved skeletal health, a reduction in adiposity and reduction in cardiometabolic risk,^1^ as well as positive psychological and social health outcomes.^2^ Considering the global trend of declining physical activity among school‐going adolescents aged 11 to 17,^3^ there is a need to develop strategies to increase physical activity among this cohort and maximise the health benefits participation delivers. Addressing this need may not only convey health benefits at the individual level, but may also contribute to a reduction in the global burden of noncommunicable disease over time, as healthy behaviours—including physical activity—in adolescence has been associated with increased healthy behaviours in later life.^4^ Worldwide, it has been estimated that physical inactivity is responsible for up to 8% of the world's noncommunicable disease burden.^5^

Australia's national sport policy, *Sport 2030*, aims to increase sport participation across the lifespan but also emphasizes the importance of physical activity in childhood and the school years to establish foundations of good health for life.^6^ *Sport 2030* defines sport broadly to include unstructured physical activity such as walking, traditional sports such as basketball and tennis, and ‘evolving’ sports such as mixed martial arts or other recreational activities.^6^, ^7^ A key priority identified in *Sport 2030* is to create a diverse sports sector on the basis that availability of varied sport options is more likely to get more individuals active.^6^ Although this priority is designed for all ages, studies in which adolescents were asked for their views on physical activity engagement confirms this as viable strategy for the adolescent cohort, as providing choice and a variety of activities was recommended to facilitate youth engagement in physical activity and sport.^8^, ^9^, ^10^ The provision of alternative sports has also been recommended previously in the Australian sport policy context to increase physical activity specifically in adolescents aged 13 to 17 who have never connected with traditional sport.^11^The definition of ‘evolving sport’ within *Sport 2030* appears to capture several sport classifications within academic literature, including ‘alternative’, ‘lifestyle’, ‘informal’ and ‘niche’ sports. While these terms are used interchangeably in the literature and there is a lack of consensus on definitions, an emphasis on the activity being distinct from mainstream or traditional rule‐based sports is common.^12^ Notably, the definition of mainstream/nonmainstream sports is also contextual, as the popularity of sports exist on a continuum often dependent on geographic region and level of media coverage.^13^

Nevertheless, activities defined as alternative sports in academic literature, such as skateboarding, surfing and BMX riding, are typically framed in opposition to traditional sports as they are usually participant led, uncompetitive and emphasise individual skill development and mastery.^14^, ^15^ In recent years, the popularity of alternative sports has expanded as they have gained increased media attention and become increasingly organised through formal competition and organised delivery.^14^, ^16^ Lifestyle and informal sports are often included under the alternative sport umbrella, such as surfing and parkour, but represent a greater emphasis on the ‘embodied’ practice of the sport, sub‐culture, and participation outside of formalised sport structures.^15^, ^17^ Finally, the term niche sport has been used primarily in the context of sport marketing literature to define sports that are considered nonmainstream, do not appeal to a mass audience and attract limited media attention.^13^ For clarity, we will use the term alternative sport throughout this paper.

Existing research on alternative sport and adolescents includes a focus on the idea of ‘sport for development’.^15^, ^18^ Alternative sports often have strong roots in subculture that emphasises the social and political context in which the sport is enacted, making them particularly relevant to adolescents deemed vulnerable, socially disadvantaged, or ‘at‐risk’.^15^, ^18^ The strong presence of subculture in alternative sports, such as the relationship between skateboarding and the counter‐culture movement,^14^ is thought to offer significant opportunities for health and personal development in marginalised groups as social bonds are formed through a ‘shared common ethos’.^14^ Individual self‐realisation may also be fostered as alternative sport participants tend to have ‘stable and shared’ forms of identity.^17^

More broadly, adolescent participation in lifestyle sports is associated with higher levels of out‐of‐school physical activity.^19^ Literature indicates that social aspects of alternative sports can confer positive psychological benefits,^20^ and lead to increased engagement amongst individuals who do not usually participate in physical activity.^12^ Where this occurs, notions of community, belonging and identity function to increase participation in physical activity *indirectly*, as young people appear to engage in alternative sports for the sense of community afforded, rather than to intentionally exercise or be active.^12^, ^21^ For example, Grabowski and Thomsen^21^ illustrate how participation in parkour offers an opportunity for changes in the way young people relate to health and physical exercise, thereby providing opportunities for lifestyle change as well as social inclusion.

Participation in ‘high‐risk’ alternative sports can also offer health benefits. Dumas and Laforest^20^ highlight how self‐evaluation of one's limits following injury in skateboarding may confer an ‘ethos of prevention’ through greater awareness of the body and health. Roberts et al^22^ suggest that participation in mountain‐biking can contribute to increased self‐esteem, in part due to the opportunity to navigate danger and overcome challenges. Finally, literature indicates that participation in martial arts and combat sports, sometimes classified as niche sports,^13^ appears to contribute to psychological well‐being.^23^

In the context of the *Sport 2030* priority to create a diverse sports sector, and the individual and public health benefits of increased physical activity, further research is required to understand how alternative sports can engage more adolescents to participate in sport. Currently, alternative interventions to increase sports participation are largely delivered within the Sport Education Model (SEM). SEM is a school‐based curriculum model designed to expose students to all aspects of sport such as field set‐up, score keeping, and refereeing.^24^ SEM has been shown to result in increased intrinsic motivation to participate,^24^ and increased feelings of relatedness specifically in students who lack motivation in sport.^25^ However, SEM still operates within a traditional sports framework.

In this exploratory paper, we seek to contribute to an evidence base of alternative sport options that can get more adolescents more active. Our aim is to understand why Australian current and past fencers decided to participate in the sport of fencing when aged under 18 years, to ascertain if fencing is a viable avenue for future program design in sports‐based health promotion for adolescents. The primary objective of this study was to identify reasons for participating in fencing. Secondary objectives were to describe and compare the frequency of participation in sport before and after experiencing fencing, and to describe and compare the levels of enjoyment between participation in sport broadly and fencing specifically.

Fencing has been chosen as the focus of this study as no literature currently exists on why individuals decide to participate—existing literature on fencing focuses primarily on the biomechanics of the sport and associated injury,^26^, ^27^ or impacts on cognition.^28^, ^29^ We have framed fencing as a sport at the intersection of the alternative and traditional domains, as although fencing has grown in popularity globally and is accessible as a competitive and organised sport,^27^ it remains nonmainstream as a lesser‐known sport within Australia. This also appears to satisfy the niche sport criteria. In Australia, the estimated proportion of children who participated in fencing in 2019 was 0.1%, while estimated child proportions for the top three sports were swimming = 36.3%, football/soccer = 15.3% and gymnastics = 11.2%.^30^

In addition, the strong emphasis on psychological factors in fencing such as intelligence, attention and perception,^29^ combined with the high demand for muscular coordination and asymmetrical movement,^26^ echoes alternative sport elements as the mode of play requires skill development and mastery rather than physical ability alone.^15^ As such, we hypothesise that this sport may provide a unique opportunity to engage adolescents that is not afforded by traditional school sports and therefore be utilised in health promotion efforts.

## 2 METHOD

### 2.1 Design

A retrospective mixed‐methods survey design was employed to identify current and former fencers' reasons for starting fencing when aged under 18 years. The survey was designed to collect qualitative data on reasons for starting fencing through free‐text responses. To answer the secondary objectives, quantitative questions were included regarding the frequency of participation in sports and fencing, and level of enjoyment of fencing and sport more broadly. These questions were designed as preliminary indicators to support our hypothesis that fencing may engage adolescents and promote physical activity participation.

The minimum sample size for this study was determined using participation rates from the AusPlay by Sport Data Table—January 2019 to December 2019.^30^ Data from 2019 were used due to the impact of the COVID‐19 pandemic on sport participation. As child participation data for fencing is noted as too unreliable to use, we used the adult participation data which, although a reliable estimate, still had a high margin of error. The estimate for adult fencers is a total population of 9500 (95% confidence interval: 2300‐16 700). Using the adult estimate n = 9500, with a 10% margin of error and 95% confidence level, a sample size of n = 96 was needed to detect significance. The target sample size of 96 also satisfies the recommended sample size for qualitative survey data between 30 and 100 responses.^31^

### 2.2 Participants

Eligible participants were considered to have started fencing within Australia when aged <18 years. They could be any age at the time of survey completion (participants aged <18 years required a parent/guardian to complete the survey on their behalf). Individuals who started fencing outside of Australia were excluded to control for the difference in popularity of fencing across countries and the influence this may have had on the decision to participate.

### 2.3 Materials

The survey consisted of 26 questions (see Appendix S1A), with skip‐logic determining which questions were made available to participants depending on their responses. Three pre‐screening questions assessed for eligibility, with the participant not being able to progress through the survey if any of the exclusion criteria were selected: aged under 18 without a parent/guardian present, began fencing when aged 18 years or over, and began fencing overseas. This paper reports on the qualitative data captured in responses to Questions 11, 12, 13 and 26 of the survey to understand reasons for participation.

Questions regarding reasons for participation were answered using free‐text. An additional checkbox‐response question was adapted from the ‘top motivations for participation’ reported in the AusPlay National Data Tables—January 2019 to December 2019^32^ by removing factors not relevant to fencing, such as ‘walk the dog’ and ‘way of getting around’, and including two options relating to alternative sports—'to try something different/alternative/nonmainstream’ and ‘to master a skill or technique’.^15^

Questions about sport participation frequency, and enjoyment of fencing and other sports, were answered on 5‐point Likert‐type scales developed by the research team for this study as preliminary indicators if fencing is enjoyable and encourages physical activity. These findings are presented in the supplement to this paper as Appendices S1B and S1C.

Prior to implementation, feedback on the survey was sought from Australian fencers (n = 2) who met one of the two inclusion criteria; started fencing when aged under 18 (n = 1) and started fencing within Australia (n = 1). One change was suggested to include a question relating to what appealed to a participant about fencing compared to other sports, but not incorporated into the survey as the research team felt that this was already captured in the existing questions. As such, no changes were made following piloting.

### 2.4 Procedure

Potential participants were recruited via email invitation sent on behalf of the research team by either the relevant state fencing association (n = 4) or local fencing club (n = 23) to their affiliated member lists. Participants were also recruited via social media with posts shared between peer fencers and in relevant fencing Facebook groups. As an incentive, participants who completed the survey could elect to enter a prize draw to win one of eight $25 (AUD) Leon‐Paul e‐gift vouchers.

The survey was administered online using the Qualtrics online survey platform (Qualtrics 2005, Version May 2021) and took on average 17 min to complete. Participant consent was implied by the completion of the survey.

### 2.5 Data analysis

Responses with invalid postcodes and answers were first removed from the dataset. Descriptive statistics were used to determine participant demographics. Postcodes of where a participant started fencing were used to calculate Socio‐Economic Indexes for Areas status through the Index of Relative Socio‐economic Disadvantage (IRSD),^33^ and remoteness area through the Australian Statistical Geography Standard Volume 5—Remoteness Structure.^34^

Stata IC 16 (StataCorp, 2019) was used for analysis of quantitative data, presented in the supplement to this paper. Normality was determined by plotting histograms and box plots, and assessing skewness and kurtosis values. Nonparametric tests of hypotheses were used as the data were not normally distributed. Wilcoxon rank‐sum tests were used to assess the median difference in enjoyment scores between genders. Wilcoxon signed‐rank tests were used to assess the difference in the median level of enjoyment and median rate of sport participation before and after fencing. A *p* < .001 was considered to indicate very strong evidence that the null hypothesis could be rejected and a value of .001 to <.01 to indicate strong evidence.

**FIGURE 1:**
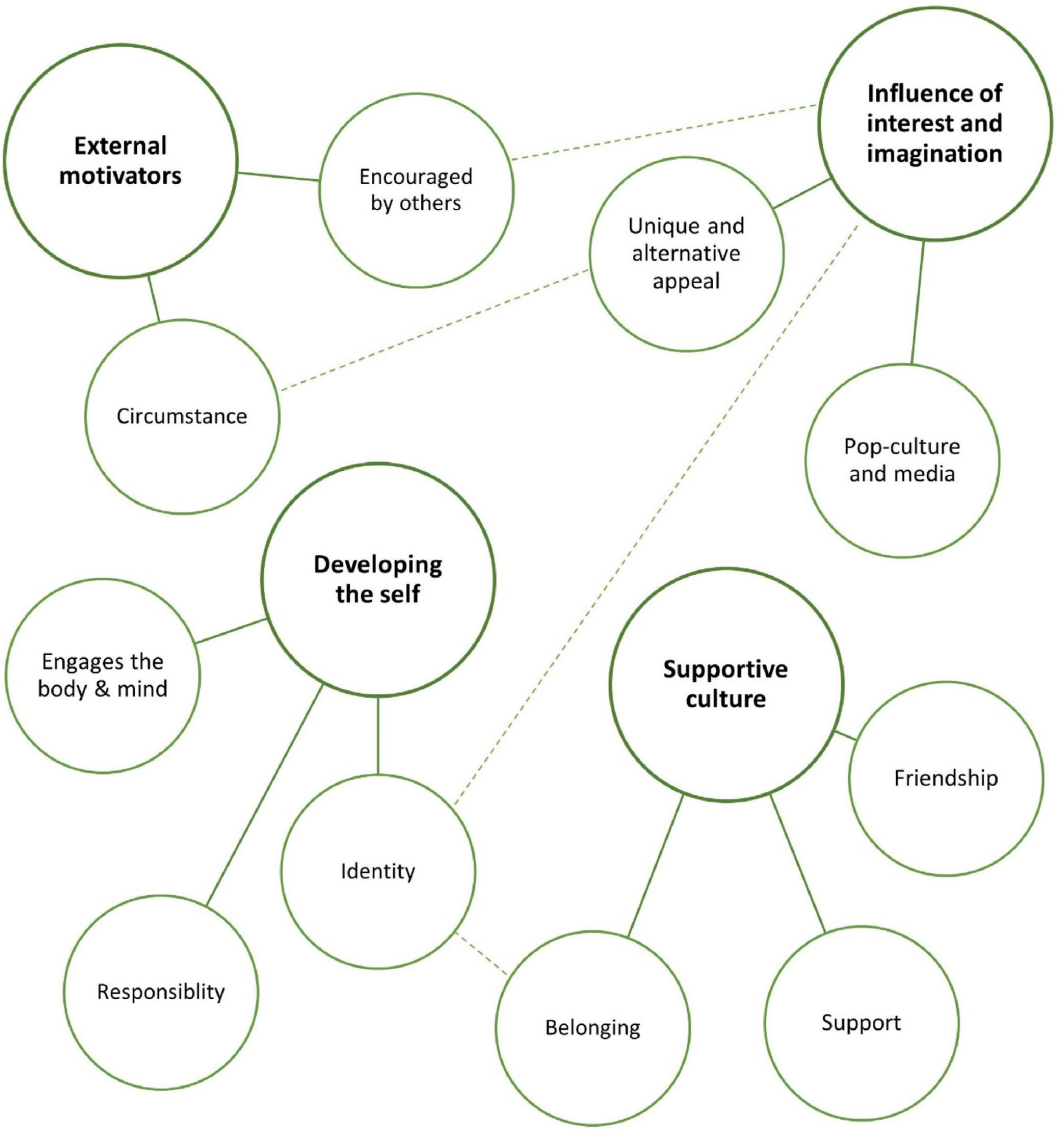
Thematic map

Qualitative findings were analysed using the six‐phase process defined by Braun and Clarke.^35^ Two researchers independently coded the entire dataset and discussed their approach to interpretation, following which codes were refined where required. While Braun and Clarke's approach to thematic analysis does not endorse the use of inter‐relater reliability as a measure of quality,^36^ dual coding was included in this study to strengthen reflexivity as one of the researchers had prior experience as a fencer and was likely to approach the data with a different understanding to someone who had not participated in the sport.^37^ A semantic and inductive approach to coding was adopted in which descriptive codes were assigned based on the raw data.^35^ Codes were first grouped into potential themes according to the respective survey question, following which themes were refined into final themes. The potential and final themes are depicted as a thematic map in Figure 1.

We approached this from a critical realist perspective, acknowledging that participant experiences are shaped by and exist within broader social contexts.^31^ This is a pertinent understanding to bring to this research as sport participation is not only based on individual psychology and preference, but also influenced by social factors such as inequity in opportunity.^38^

## 3 RESULTS

A total of 163 participants attempted to begin the survey, but only 101 valid responses were received and included in analysis due to not meeting the eligibility criteria or providing incoherent responses.

Of the 101 valid responses received, the mean number of years fenced was 9.9 years (SD = 8.38), median age group was 18 to 24 years, and median age group when started fencing was 12 to 14 years. Participant characteristics are detailed in Table 1.

**TABLE 1:** Participant characteristics

	n	Percentage
Participants	101	100.0
Gender		
Female	38	37.6
Male	63	62.4
Age group in years when started fencing		
0‐4	1	1.0
5‐8	8	7.9
9‐11	28	27.7
12‐14	48	47.5
15‐17	16	15.8
Age group in years		
9‐11	1	1.0
12‐14	13	12.9
15‐17	21	20.8
18‐24	32	31.7
25‐34	18	17.8
35‐44	6	5.9
45‐54	8	7.9
55‐64	2	2.0
State		
ACT	8	7.9
NSW	28	27.7
QLD	15	14.9
SA	6	5.9
TAS	2	2.0
VIC	28	27.7
WA	14	13.9
IRSD decile^a^		
Deciles 1‐3 (most disadvantage)	3	3.0
Deciles 4‐7	29	28.7
Deciles 8‐10 (least disadvantage)	69	68.3
Remoteness		
Major cities of Australia	84	83.2
Inner regional Australia	7	6.9
Inconclusive^b^	10	9.9
Weapons fenced		
Sabre	12	11.9
Epee	15	14.9
Foil	32	31.7
Multiple	42	41.6
Club context		
Community/local club	62	61.4
School	9	8.9
Both	30	29.7

^a^ IRSD deciles are ranked from 1 to 10, with a low score indicating relatively greater disadvantage and a high score indicating a relative lack of disadvantage.^33^

^b^ Postcode included more than one remoteness area.

In the checkbox‐response question on motivating factors, presented in Table 2, fun/enjoyment was selected as the most common factor considered in the decision to start fencing, followed by the desire to try something different/alternative/nonmainstream. To master a skill or technique was the sixth most common factor.

**TABLE 2:** Motivating factors

	n	Percentage
Motivating factors		
Fun/enjoyment	79	24.2%
To try something different/alternative/nonmainstream	67	20.6%
To learn and develop a new skill	49	15.0%
To get fit/keep fit	35	10.7%
Performance/competition opportunities	29	8.9%
To master a skill or technique	27	8.3%
Social reasons	19	5.8%
Other^a^	11	3.4%
Psychological/mental health benefits	10	3.1%
None of the above	3	0.9%
Total^b^	326	100%

^a^ Reasons cited under the ‘other’ category included being required by school to choose a sport (n = 1), the indoor setting in which fencing is practiced (n = 1), an interest in history and weaponry (n = 3), not being a ball or team sport (n = 1), opportunities to travel (n = 1), feeling drawn to the sport (n = 1), the appeal of sword fighting and mastery in swordsmanship (n = 2) and liking Star Wars (n = 1).

^b^ Responses do not add up to 101 as participants were able to select multiple factors.

Four themes were generated about why a participant decided to start fencing and what they liked about fencing compared to other sports: external motivators, influence of interest and imagination, the opportunity to develop the self and a supportive culture.

### 3.1 External motivators

Participants reported that the decision to start fencing is often initiated by something or someone external to the self. External motivators primarily included receiving encouragement from others to start fencing, and the circumstantial requirement to participate in a winter school sport at school.

Many participants started fencing after being encouraged by a family member, friend or other person known to them. Some participants suggested that the recommendation from another was based on a judgement that the characteristics of fencing would be particularly valued by, or suited to, the individual. For example, one participant had received a recommendation from a doctor to pursue fencing as this was deemed beneficial to strengthen her weak ankles.

*I had weak ankles when I was younger…and a doctor recommended something like ballet or fencing. My mum found a club and helped introduce it to me* (P68, aged 18‐24, female).

Other participants were encouraged by their parents on the assumption that they would enjoy the sport.

*My mother took me to a local club because she thought it would be something that I would enjoy* (P61, aged 18‐24, female).

*I was encouraged to start fencing by my parents in primary school. They were keen for me to be active across multiple sports and thought that fencing might be more interesting than some other sports on offer at my school* (P3, aged 25‐34, female).

For participants who were influenced by circumstance, the decision to start fencing included a greater element of passivity as the sport was already known to them and did not need to be actively sought out. For many, this was due to a requirement of their school curriculum to participate in a winter sport—a finding was consistent with participant characteristics, as over one third of participants started fencing through school. For some participants who were required to choose a winter sport, fencing was selected as it appeared to be the most interesting option available compared to other sports.

*I was required to choose a winter sport at school, and chose fencing both because I was interested in it, but also because it was indoors…* (P89, aged 18‐24, male).

*Part of our high school program involves choosing a winter sport to participate in. At the time, fencing seemed like the most interesting choice* (P73, aged 18‐24, male).

*Wednesday afternoon school elective, and fencing seemed the best option of netball, football* (P101, aged 12‐14, female).

Other external motivators less frequently noted, but nonetheless indicative of a significant concept, included consideration of health and safety in sport participation. This was reported in the context of longevity and physical safety,

*Sibling already doing fencing, good sport can last lifelong, lower risk of serious injury compared to traditional Australian sports* (P38, aged 15‐17, male).

as well as an emphasis on the indoor setting.

*Environment (indoors) is appealing/safe* (P82, aged 12‐14, female).

A handful of participants framed the importance of the indoor setting in terms of shelter afforded from the Australian sun and heat.

*My dad wanted us to do it as he thought it was cool and a good opportunity to do a sport indoors. He had been a professional junior tennis player and did not want us to have to deal with skin cancers…* (P81, aged 18‐24, female).

*I do also enjoy that it is almost always indoor (Air conditioning, yay)* (P68, aged 18‐24, female).

### 3.2 Influence of interest and imagination

For many participants the desire to explore one's interests and imagination was central in their decision to start fencing. For example, participants reported actively seeking out fencing after seeing representations of sword fighting in popular culture and media, such as films and literature, or having an interest in military history.

*I read a lot of fantasy books and wanted to try sword fighting* (P41, aged 35‐44, female).

*Interest in sword fighting motivated by movies (e.g. Pirates of the Caribbean)* (P1, aged 25‐34, female).

*Interest in military history, specifically the history of combat and arms* (P16, aged 25‐34, male).

The desire to try something unique and alternative, the second most common factor considered when deciding to start fencing (Table 2), also emerged as a key element of this theme. Many participants noted that the uniqueness and apparent ‘coolness’ of fencing had sparked their interest and was a reason for starting fencing.

*I decided to start fencing due to the uniqueness of the sport. I personally did a project on it in Primary School and found the sport really interesting* (P98, aged 12‐14, male).

Assumptions that fencing was a cool and unique sport appeared to be influenced by media representation–

*Because it's cool, and I had seen it on the Parent Trap when I was younger*. (P67, 18–24, female).

–the appeal of swords, and for some participants, a discontent with traditional sports. For example, participants reported turning to fencing after no longer wanting to participate in swimming and gymnastics.

*I was doing gymnastics but did not want to do that anymore and so mum said I had to try a different sport, so I decided I would like to try fencing after seeing it in “The Parent Trap”* (P29, female, 25‐34).

*Did swimming and got bored of it, so fencing looked cool with the swords…* (P94, aged 15‐17, male).

In addition, the use of an actual sword in fencing and associations with the imaginative emerged as a kind of ‘sword appeal’ across some responses. For a few participants, this was connected to memories of childhood play and a long‐held interest in sword fighting.

*Enjoyed sword fighting with sticks, saw a demo at a medieval fair and signed up* (P58, aged 12‐14, male).

*Loved swordfighting as a child* (P63, aged 45‐54, female).

### 3.3 Developing the self

Many participants actively sought out fencing and found it appealing because the unique characteristics of the sport encouraged personal development—where personal development is understood in this context as being stimulated, challenged and afforded an opportunity to learn. Overwhelmingly, fencing was framed as a sport that ‘has it all’ in that is both physically and mentally challenging, engaging both the body and mind.

*It's all in one: fitness, strength, technique, strategy, competition. For me it's like meditation ‐ cannot think about anything else but fencing when you are doing it* (P9, aged 45‐54, female).

*…I also love how it is a really unique combination of skills ‐ coordination, speed, balance, and there is such a strong mental aspect required as well (to anticipate opponent's moves and try to outsmart them and think about your strategy and what moves you should make). I've never played another sport with that kind of skillset, because of this unique combination I find it more engaging than other sports* (P97, aged 18‐24, female).

While participants noted that fencing was great for fitness, the appeal of the mental aspects of fencing that require attention to strategy and tactics was reported more prevalently as a drawcard over physical benefits.

*Fencing has a greater focus on mental aspects than other sports. While it is physically demanding, the most enjoyable element is the process of trying new things, figuring out how to win points, and keeping an even keel…* (P89, aged 18‐24, male).

For some participants, the emphasis on strategy presented an opportunity to take ownership of their experience, affording a strong sense of autonomy in the sport.

*I like how it is extremely technical but that there is also a lot of freedom with how I can fence as an individual when presented with different situations in a bout…* (P81, aged 18‐24, female).

*…I like the strong learning curve as you need to learn priority which is completely counter intuitive in many ways. Furthermore, the rules are very deep and nuanced which allows for individuals to bring their own style and application of the rules to the post…* (P33, aged 18‐24, male).

Others placed a strong emphasis on the individual nature of fencing, and more specifically, the idea of individual responsibility which afforded an opportunity for growth,

*It is not a team sport or luck based, if I lose it is always because I was the worse fencer, and it's always on me. I think this really helps me to improve in fencing and outside of it* (P77, aged 15‐17, male).

*It's the individual that makes the difference it all relies on your performance* (P66, aged 15‐17, male).

*There is a team element and a strong sense of community stemming from it however the sport itself is rather individualised in and of itself. In a team sport you either compensate for what another lacks or someone does the same for you. When you fail the team fails. For the most part in fencing the only person you let down is yourself. However, when you succeed there is a community to help elevate you when you learn and win and better yourself* (P95, aged 18‐24, male).

Finally, a handful of participants described a deeper level of personal development, as they suggested that participation in fencing had an influence on their sense of identity and self‐understanding. For example, one participant noted how learning to navigate the changing circumstances of winning and losing contributed to their confidence and independence.

*It challenged me, and was always changing. You could beat someone one day and lose to them the next. It taught me how to be brave, independent and be my own knight in shining armour* (P26, aged 18‐24, female).

Another participant reported the ongoing potential for self‐exploration as a reason for continued participation.

*…once I started sabre it felt like I connected with parts of myself I had not explored and really enjoyed it* (P96, aged 18‐24, male).

### 3.4 Supportive culture

This theme highlights the reported sense of community and apparent ‘shared ethos’ amongst fencers, that helps to foster experiences of belonging and friendship for participants. Often, the sense of belonging afforded by fencing was framed in contrast to traditional sports that may exclude individuals based on limited criteria for success. For example, participants stated that the physical attributes and skills typically required for success in traditional sports were not as relevant to fencing—there were other ways to find success, particularly through strategy, which allowed people of all skill levels to participate.

*You do not have to be big and strong and “tough”…* (P25, aged 15‐17, male).

*…I also like that your physical attributes are not as important as in other sports, you can be any body shape or height and be good at fencing, whereas for other sports like athletics or swimming or gymnastics, it is not the same* (P29, aged 25‐34, female).

*I like the fact that it's accessible regardless of gender, age, and to a greater degree than a lot of sports, physical ability. It's also a sport you can play quite seriously well into adulthood (this wasn't something that motivated me to start, it's just something I've noticed)* (P31, aged 25‐34, female).

The idea that fencing affords a space for people who may not have been able to or interested in participating in traditional sports was also evident amongst responses that highlighted the relationship between identity and sport participation. For example, some participants noted that fencing provides a space for belonging for people who do not fit in or identify as being ‘nonmainstream’.

*…it's a sport in which non‐mainstream people seem to thrive* (P6, aged 45‐54, female).

*Fencing is fantastic for people who find they do not fit in conventional sports…* (P29, aged 25‐34, female).

Further to this, a number of participants valued the strong sense of community and support offered in fencing, attributed to the fact that fencing brings people together with similar interests.

*The other boys at the club have similar interests to me and aren't just interested in AFL and cricket* (P58, aged 12‐14, male).

*Everyone is more relaxed at a club level and have similar interests* (P23, aged 18‐24, female).

*…great community at club level, lots of like‐minded people…* (P60, aged 18‐24, female).

Some attributed community support to the fact that the sport is small in Australia. Fencers can easily get to know one another through competition at both the state and national level, fostering greater opportunities for relationship building.

*Fencing is a small community and I was heavily encouraged through great personal relationships. My coach always spoke to me about my own goals and journey, and I had teammates who mentored me (and even travelled with me when I first started)*. (P44, aged 18‐24, male).

*…the fencing community is very welcoming, and a nice group to be a part of, due in part to its smaller size* (P89, aged 18‐24, male).

However, the ‘smallness’ of the sport was also cited as a barrier to access depending on location. One participant noted that in their state it was difficult to find others to train and interact with.

*I really like fencing but it is difficult being part of a sport with so few people to train with, interact with and to volunteer and be on committees in my state compared to some other states* (P81, aged 18‐24, female).

Another participant indicated that gender and socio‐economic status presented a barrier to fencing due to the smallness of the sport in Australia which receives limited funding, and is less available in all‐girls schools.

*Fencing is awesome but expensive and Australia is not funded well enough in the sport. Also it should be encouraged more at girls schools not just boys schools* (P91, aged 18‐24, female).

## 4 DISCUSSION

The aim of this study was to understand why young Australians decide to participate in the sport of fencing, to determine if fencing is a useful avenue for sports‐based health promotion in adolescents. This is, to our knowledge, the first study investigating this topic and our inquiry was therefore exploratory in nature.

We acknowledge that adolescents often start sports for reasons other than wanting to be physically active, such as for fun and to improve skills,^39^ and that ‘traditional’ sports can satisfy the needs of adolescents when delivered in different formats such as the SEM,^24^ so the findings presented below are not explicitly unique to fencing. However, it has been suggested that novelty may be critical in eliciting and sustaining participation,^40^ and so increasing the variety of sport options available to adolescents may be beneficial. Our findings show that an element of novelty is afforded within fencing via the opportunity for continued learning and mental challenge, as cited by participants, positioning fencing as a sport that could facilitate ongoing interest and thereby participation.

This is most apparent within the ‘developing the self’ theme, which indicates that the degree of strategy and skill required in fencing, rather than physical ability alone, is a key drawcard in attracting participants to the sport. This aspect is beneficial in that it affords participants the opportunity to learn and challenge themselves both physically and mentally, an element that echoes findings in martial arts literature.^23^ The appreciation of the challenge presented by fencing also echoes findings in alternative sport literature in which participants value opportunities to overcome physical and emotional obstacles as they adapt to environmental challenges.^18^ In this case, the appreciation for the unpredictability of the environment appears to be exchanged for an appreciation of the unpredictability of an opponent. This may also be understood as an opportunity for resilience building—an element identified in relation to adolescent participation in nonsport activities such as circus arts, as fencers must learn to overcome failure when losing a bout.^41^

While the practice of fencing is inherently combative as one opponent competes against the other, participants expressed an appreciation for the opportunity to learn and be creative as they try new skills during bouts, emphasising the self‐development aspect of participation rather than competition per se (although there were some exceptions). Parallels to alternative sport literature are evident here, as although alternative sport practitioners often attribute their enjoyment to the fact that their activities are noncompetitive, the focus on individual skill development is highly valued.^12^, ^15^, ^42^ Furthermore, Gilchrist and Wheaton^12^ highlight how masculine identity amongst male parkour practitioners appears less tied to ‘hegemonic masculinity’ as typically ‘feminine’ skills such as balance and agility are valued. While this study did not explore gendered perceptions of fencing, the finding that participants do not feel that they need to be ‘big and strong and tough’ indicates potential for changing gender stereotypes associated with sports participation.

With respect to the ‘supportive culture’ theme, our finding of the opportunity for belonging and relatedness with others through fencing is indicative of elements of a sub‐culture at play. In reference to the sub‐cultural characteristics of parkour, Grabowski and Thomsen^21^ attribute group identification and identity construction to the fact that practitioners are both ‘individually unique’ and ‘collectively accepted’—factors which evoke our finding that fencing is a place for nonmainstream people to thrive and the coming together of people with similar interests. As the establishment of social bonds in alternative sport participation is shown to create an environment conducive to sustained sports participation,^14^, ^17^ this finding suggests that fencing may be a useful tool to appeal to adolescents disengaged by traditional sports or those seeking new ways to explore their relationship to movement more broadly,^21^ especially as participants felt that the supportive culture of fencing provided a space to enjoy sport for those who do not have the ‘typical’ physical attributes that traditional sport participation demands. Further to this, evidence shows an association between adolescent sport dropout and perceptions of physical and sport competence.^43^ As participation in fencing may offer a new sense of physical ability and competence in participants who do not possess attributes required for traditional sport, this supports the notion that fencing could offer an opportunity for lasting participation amongst adolescents. However, as our study investigated fencing participation in a broad cohort of participants, the majority of whom had already participated in sports prior to fencing, further research is required to confirm this.

Finally, key reasons for participation in fencing are captured in both the ‘external motivators’ theme as well as ‘influence of interest and imagination’ theme. Findings within the latter indicate that the representation of sword fighting in pop‐culture is a key motivator for participation. This finding aligns with niche sport literature on the role of curiosity and interest in sport involvement.^44^ In relation to the former, as encouragement from others, including parents, was identified as a key external motivator, this study reinforces findings in literature that support from family is a key facilitator of youth participation in sport.^45^ Also of value is the longevity and safety of fencing in that it can be done at any age, which presents an opportunity for participation across generations. Studies show that some youth may prefer sports that they can do with, or in the presence of, their family members,^8^, ^46^ which will be an asset to any future fencing‐based health promotion intervention.

Overall, this exploratory study provides evidence that fencing has valuable characteristics that may engage adolescents in physical activity.

### 4.1 Directions for future research

Significantly, the findings of this exploratory paper have presented a number of directions for future research. Across all our themes aside from external motivators, it appeared that intrinsic motivation played a part in the decision to start fencing, that is, participants exhibited a tendency to seek out challenges, develop one's capacities, learn and explore, and to participate out of genuine interest rather than due to external pressures.^47^ As those who are intrinsically motivated tend to be more engaged in sport,^24^ this positions fencing as a viable avenue for sports‐based health promotion. We recommend future research to better understand how intrinsic motivation operates in this space, and whether fencing is particularly valuable in appealing to ‘amotivated’ adolescents because of its apparent ability to satisfy psychological needs.^48^

Second, we suggest that access as a barrier to participation in fencing be explored, as access to the sport via school was identified as a key feature that facilitates uptake. Remoteness classification of participants indicated that the majority of fencers started within major cities of Australia, suggestive of limited access to the sport for individuals living outside of urban centres. As children are more likely to participate in sport via a sports club or association in Australia,^7^ improving access to local clubs is an obvious direction to address the discrepancy in participation based on location. However, these barriers may be better addressed by prioritising school‐based programs to access fencing, as schools have been identified as potential settings to address inequity in physical activity.^49^ Research is needed to identify settings that can best improve access to fencing among adolescents and associated barriers to its provision.

Notably, the indoor setting of fencing stood out as a unique benefit in the context of the Australian summer and risk of skin cancer. However, sports practiced indoors and sports that require expensive equipment may be prohibitive to individuals with lower socio‐economic status,^50^ thus compounding equity‐based differences in sport participation. Alternative sports such as surfing have also been identified as more costly to implement than traditional sports due to the equipment and transport costs.^18^ While most fencing clubs provide equipment for use if participants do not wish to or cannot purchase their own, as most participants started fencing in an area of least disadvantage (IRSD deciles 8‐10), this may indicate existing inequity in access to the sport based on socio‐economic status. Research into alternative and cost‐effective sport delivery models, such as through school programs, sponsorships^†^ or state‐based ‘active children’ rebate schemes,^‡^ is therefore required to determine the viability of fencing as an accessible sports‐based health promotion intervention. As a preliminary indication, the estimated cost of fencing per state is presented in Appendix S1D.

### 4.2 Limitations

While the target sample size was achieved, limitations in sampling result from the use of a convenience approach, and the over‐representation of some states in Australia compared to others. Results therefore may not be representative of all fencers in Australia, and include an element of selection bias as participants self‐selected to complete the survey. Recall bias may also be present, reflecting a range of time duration between when participants started fencing when aged under 18 years and the time of the survey. This was a deliberate choice to capture the views of as many fencers as possible. A limitation of the methodology is that the survey tool was developed by the research team for this exploratory study and did not utilise validated measures. Finally, the positionality of one author as a former fencer is both a limitation and strength in that this former experience affords an understanding of how the sport is practiced, which has impacted and assisted interpretation of the data.

## 5 CONCLUSION

Our findings suggest that fencing may be a viable sports‐based health promotion intervention to increase adolescent participation in physical activity broadly, as participants were drawn to the sport because of the opportunity to explore their interests and develop their skills in a supportive setting. As this is the first known study exploring why individuals participate in fencing during adolescence, further research is required to understand fencing's potential in different socio‐economic and geographic contexts, and across different cohorts of adolescents, to determine facilitators and barriers to access on a wider scale.

## CONFLICT OF INTEREST

The authors declare no conflicts of interest.

## ETHICS STATEMENT

The study received ethical approval from the University of New South Wales Human Research Ethics Advisory Panel (HC210216).

## Supporting information


**Appendix S1** Supporting Information

## Data Availability

The data that support the findings of this study are available on request from the corresponding author. The data are not publicly available due to privacy or ethical restrictions.

## Endnotes

^†^
For example, a fencing club in Victoria is advertised as offering fencing ‘at no cost’ to immigrant children and their families through donations and sponsorships. However, information on how this delivery model is funded is not available online.

^‡^
Many fencing clubs participate in their respective state‐based rebate schemes for sport participation, which can offer up to $200 AUD off membership fees annually. For an example see https://www.service.nsw.gov.au/campaign/active-kids.
