# Supplementary material for: Exploring why young Australians participate in the sport of fencing: Future avenues for sports‐based health promotion
Source: Health Promot J Austr. 2022 Aug 30;34(1):48–59. doi: 10.1002/hpja.650 (PMC10087125; doi:10.1002/hpja.650)
Supplement: Supplementary file 1 — Appendix S1 Supporting Information [file HPJA-34-48-s001.pdf]

SUPPLEMENT

APPENDIX A

Survey tool

*Pre-screening**

1. I am: Under 18 years of age ☐ A parent/guardian completing this survey for a child under 18 years of age ☐ Over 18 years of age ☐
2. Age when started fencing: 0-4 ☐ 5-8 ☐ 9-11 ☐ 12-14 ☐ 15-17 ☐ 18+ ☐
3. I started fencing: In Australia ☐ Overseas ☐

Basic information

4. Age (current): 0-4 ☐ 5-8 ☐ 9-11 ☐ 12-14 ☐ 15-17 ☐ 18-24 ☐ 25-34 ☐ 35-44 ☐ 45-54 ☐ 55-64 ☐ 65+ ☐
5. Gender: Male ☐ Female ☐ Non-binary ☐ Prefer not to say ☐
6. Postcode (current) (Free format response (FFR))
7. Postcode (when you started fencing) (FFR)
8. Years spent fencing (FFR)
9. Club context: School ☐ Community/local club ☐
10. Weapons fenced: Sabre ☐ Epee ☐ Foil ☐

Reasons for starting

11. Why did you decide to start fencing? (FFR)
12. Did you consider any of the following factors when you decided to start fencing? You can select multiple: To try something different / alternative / non-mainstream ☐ To learn and develop a new skill ☐ To master a skill or technique ☐ Fun / enjoyment ☐ Social reasons ☐ To get fit / keep fit ☐ Psychological / mental health benefits ☐ Performance / competition opportunities ☐ None of the above ☐ Other (FFR)
13. What do you like about fencing compared to other sports? (FFR)

Enjoyment indicators

14. How much did you enjoy sport or physical activity before you started fencing? A great deal ☐ A lot ☐ A moderate amount ☐ A little ☐ None at all ☐
15. How much did you / do you enjoy fencing? A great deal ☐ A lot ☐ A moderate amount ☐ A little ☐ None at all ☐
16. How much do you enjoy sport or physical activity since starting fencing? A great deal ☐ A lot ☐ A moderate amount ☐ A little ☐ None at all ☐

Physical activity indicators

17. Did you participate in any sport or physical activity before you started fencing? Not including compulsory school sport of P.E. classes. Yes ☐ No ☐ (if no is selected, the following two questions are skipped).
18. What sports or physical activity did you participate in? (FFR)
19. How often did you participate in sport of physical activity before you stated fencing? Not including compulsory school sport of P.E. classes: Once a week ☐ Twice a week ☐ Three times a week ☐ Four times a week ☐ Five or more times a week ☐
20. How often did you participate in fencing when you first started? Once a week ☐ Twice a week ☐ Three times a week ☐ Four times a week ☐ Five or more times a week ☐
21. Did the time you spent fencing per week increase the longer you were involved in the sport? Yes ☐ No ☐ (if no is selected the following question is skipped).

22. What did this increase to? Twice a week ☐ Three times a week ☐ Four times a week ☐ Five or more times a week ☐
23. Did you participate in any sport or physical activity after you started fencing (not including fencing)? Yes ☐ No ☐ (if no is selected the following two questions are skipped).
24. What sports or physical activities did you participate in after you stared fencing (not including fencing)? (FFR)
25. How often did you participate in other sports or physical activity after you started fencing? Once a week ☐ Twice a week ☐ Three times a week ☐ Four times a week ☐ Five or more times a week ☐

Final question

26. Are there any other comments you would like to add about why you started fencing or why you like it? (FFR)

*Pre-screening questions: If a participant selected 'I am = under 18 years of age', 'age when started fencing = 18+', or 'I started fencing = overseas', they would not be able to progress further.

APPENDIX B

Levels of enjoyment

There was very strong evidence ($p < 0.001$) that the median score of enjoyment of sport participation is significantly different before (3, IQR=3) and after (5, IQR=2) fencing, reflecting an increase from 'a moderate amount' to 'a great deal'. Responses are presented in Table I.

There were no significant differences in levels of enjoyment between genders. There was no evidence ($p = 0.564$; $z = 0.577$) that the median level of enjoyment of sport participation before fencing among females (4, IQR=2) was different from that of males (3, IQR=3). There was weak evidence ($p = 0.093$; $z = -1.678$) that the median level of fencing enjoyment among females (5, IQR=1) was different to that of males (5, IQR=0). There was no evidence from ($p = 0.554$; $z = -0.592$) that the median level of enjoyment of sport participation after fencing among females (4.5, IQR=2) was different to that of males (5, IQR=1).

Table I: Levels of enjoyment

**Survey item and respondent group**	**None at all *n* (%)**	**A little *n* (%)**	**A moderate amount *n* (%)**	**A lot *n* (%)**	**A great deal *n* (%)**	**Median (IQR)†**	**Total *n* (%)**
How much did you enjoy sport or physical activity before you started fencing?							
Overall	3 (3.0)	23 (22.8)	26 (25.7)	16 (15.8)	33 (32.7)	3 (3)	101 (100)
Females	2 (5.3)	7 (18.4)	9 (23.7)	6 (15.8)	14 (36.8)	4 (2)	38 (100)
Males	1 (1.6)	16 (25.4)	17 (27.0)	10 (15.9)	19 (30.2)	3 (3)	63 (100)
How much did you/do you enjoy fencing?							
Overall	1 (1.0)	0 (0.0)	2 (2.0)	20 (19.8)	78 (77.2)	5 (0)	101 (100)
Females	1 (2.6)	0 (0.0)	1 (2.6)	10 (26.3)	26 (68.4)	5 (1)	38 (100)
Males	0 (0.0)	0 (0.0)	1 (1.6)	10 (15.9)	52 (82.5)	5 (0)	63 (100)
How much do you enjoy sport or physical activity since starting fencing							
Overall	0 (0.0)	4 (4.0)	22 (21.8)	23 (22.8)	52 (51.5)	5 (2)	101 (100)
Females	0 (0.0)	2 (5.3)	10 (26.3)	7 (18.4)	19 (50.0)	4.5 (2)	38 (100)
Males	0 (0.0)	2 (3.2)	12 (19.1)	16 (25.4)	33 (52.4)	5 (1)	63 (100)

†Responses were coded 1 = not at all, 2 = a little, 3 = a moderate amount, 4 = a lot, 5 = a great deal.

APPENDIX C

Sports participation

Eighty six percent of respondents participated in sport before starting fencing, not including compulsory school sports and physical education classes. Of these respondents, only ten percent solely practiced a sport that was not listed in the top-10 most common activities for children in Australia.[1] Sixty six percent of respondents participated in other sport after starting fencing. Out of these respondents, thirty three percent solely practiced a sport not listed in the top-10 most common activities for children.[1]

The median time spent fencing per week, when a participant first started the sport, was once a week. Eighty seven percent of respondents indicated that the time spent fencing per week increased the longer they were involved in the sport. The median time this increased to was four times a week. There was very strong evidence ($p < 0.001$) that the median fencing frequency was significantly different at the start from once a week (1, IQR=1) and the longer a participant was involved in the sport to four times a week (4, IQR=3). Participation rates are detailed in Table II.

Table II: Participation proportions

**Survey item and respondent group**[†]	**Once a week *n* (%)**	**Twice a week *n* (%)**	**Three times a week *n* (%)**	**Four times a week *n* (%)**	**Five or more times a week *n* (%)**	**Median (IQR)** [§]	**Total *n* (%)**
Participation frequency in sport or physical activity before starting fencing[‡]							
Overall	23 (26.4)	31 (35.6)	19 (21.8)	9 (10.3)	5 (5.6)	2 (2)	87 (100)
Females	5 (15.2)	10 (30.3)	9 (27.3)	6 (18.2)	3 (9.1)	3 (2)	33 (100)
Males	18 (33.3)	21 (38.9)	10 (18.5)	3 (5.6)	2 (3.7)	2 (2)	54 (100)
Participation frequency in fencing – at start							
Overall	64 (63.4)	31 (30.7)	5 (5.0)	1 (1.0)	0 (0.0)	1 (1)	101 (100)
Females	25 (65.8)	13 (34.2)	0 (0.0)	0 (0.0)	0 (0.0)	1 (1)	38 (100)
Males	39 (61.9)	18 (28.6)	5 (7.9)	1 (1.6)	0 (0.0)	1 (1)	63 (100)
Participation frequency in fencing – increase[‡]							
Overall	-	25 (28.4)	17 (19.3)	16 (18.2)	30 (34.1)	4 (3)	88 (100)
Females	-	10 (31.3)	5 (15.6)	6 (18.8)	11 (34.4)	4 (3)	32 (100)
Males	-	15 (26.8)	12 (21.4)	10 (17.9)	19 (33.9)	4 (3)	56 (100)
Participation in sport or physical activity after starting fencing[‡]							
Overall	20 (29.9)	14 (20.9)	16 (23.9)	7 (10.5)	10 (14.9)	2 (3)	67 (100)
Females	6 (22.2)	6 (22.2)	7 (25.9)	5 (18.5)	3 (11.1)	3 (2)	27 (100)
Males	14 (35.0)	8 (20.0)	9 (22.5)	2 (5.0)	7 (17.5)	2 (2)	40 (100)

[†]Responses for the questions in this table do not equal 101, as questions were only made available to participants who indicated that they did participate in sport or physical activity before and after fencing, and that fencing participation frequency increased the longer they were involved in the sport.
[‡]Not including compulsory school sport of P.E. classes.
[§]Responses were coded 1 = once a week, 2 = twice a week, 3 = three times a week, 4 = four times a week, 5 = five or more times a week.

---

[1] Sport Australia. AusPlay: January 2019 to December 2019 – by sport data table [Internet]. 2020 [Accessed 8 Aug 2021]. Available from: https://www.clearinghouseforsport.gov.au/research/ausplay/results

APPENDIX D

Estimated cost of fencing

Table III: Estimated junior fencing costs per state†

State	Beginner course‡	Annual membership§
VIC	180 – 470	330 – 1090
NSW	55 – 245	140 – 1120
ACT	90 – 255	200 – 300
QLD	0 – 144	100 – 885
SA	100 – 150	400 – 660
WA	105 – 180	320 – 750
TAS	NA	140 – 350
NT	NA	NA

†Due to variations in fee structure amongst clubs, the lowest and highest annual membership option has been listed for each state. Costs are presented in $AUD, accessed on 09 July 2022.

‡Beginner courses range from 5 to 10 weeks

§Notes on memberships:

- Some clubs offer a junior and adult membership option, whereas others only offer one standard membership rate. The junior membership option has been included for clubs that offer this.
- Annual memberships generally refer to a 40-week period (a school year) and may include access to one group training session or more per week. Individual fencing lessons are typically not included in membership costs. Where clubs offer separate memberships for one or two training sessions per week, the price of a single-session membership has been included in this table.
- Affiliation with a state fencing body in addition to membership is required by some clubs.
